# AND-1 fork protection function prevents fork resection and is essential for proliferation

**DOI:** 10.1038/s41467-018-05586-7

**Published:** 2018-08-06

**Authors:** Takuya Abe, Ryotaro Kawasumi, Michele Giannattasio, Sabrina Dusi, Yui Yoshimoto, Keiji Miyata, Koyuki Umemura, Kouji Hirota, Dana Branzei

**Affiliations:** 1 IFOM, the FIRC Institute of Molecular Oncology, Via Adamello 16, 20139 Milan, Italy; 2 Department of Chemistry, Graduate School of Science, Tokyo Metropolitan University, Minamiosawa 1-1, Hachioji-shi, Tokyo, 192-0397 Japan; 3 Dipartimento di Oncologia ed Emato-Oncologia, Università degli Studi di Milano, Milan, 20122 Italy; 4 Istituto di Genetica Molecolare, Consiglio Nazionale delle Ricerche (IGM-CNR), Via Abbiategrasso 207, 27100 Pavia, Italy

## Abstract

AND-1/Ctf4 bridges the CMG helicase and DNA polymerase alpha, facilitating replication. Using an inducible degron system in avian cells, we find that AND-1 depletion is incompatible with proliferation, owing to cells accumulating in G2 with activated DNA damage checkpoint. Replication without AND-1 causes fork speed slow-down and accumulation of long single-stranded DNA (ssDNA) gaps at the replication fork junction, with these regions being converted to DNA double strand breaks (DSBs) in G2. Strikingly, resected forks and DNA damage accumulation in G2, but not fork slow-down, are reverted by treatment with mirin, an MRE11 nuclease inhibitor. Domain analysis of AND-1 further revealed that the HMG box is important for fast replication but not for proliferation, whereas conversely, the WD40 domain prevents fork resection and subsequent DSB-associated lethality. Thus, our findings uncover a fork protection function of AND-1/Ctf4 manifested via the WD40 domain that is essential for proliferation and averts genome instability.

## Abstract (web summary)

AND-1, the vertebrate orthologue of Ctf4, is a critical player during DNA replication and for maintenance of genome integrity. Here the authors use a conditional AND-1 depletion system in avian DT40 cells to reveal the consequences of the lack of AND-1 on cell proliferation and DNA replication.

## Introduction

Faithful DNA replication is essential to prevent accumulation of mutations and genome rearrangements, which are leading causes of genome instability. DNA replication is carried out by the replisome, minimally composed of the replisome progression complex (RPC) and DNA polymerases^1,2^. RPC consists of the CMG complex, comprising Cdc45, the MCM helicase, and GINS, and accessory factors that help efficient DNA replication by removing nucleosomes ahead of the replication forks, resolving DNA topological problems, and assisting in the bypass of DNA damage^1,3^.

AND-1 (acidic nucleoplasmic DNA-binding protein), a component of RPC, is a highly conserved protein with orthologs spanning from fungi to vertebrates. Its ortholog in budding yeast is known as Chromosome Transmission of Fidelity 4, Ctf4, and it was identified in screens of mutants with increased rates of mitotic chromosome loss^4^. AND-1/Ctf4 facilitates cell cycle progression, particularly in late S through G2/M^5–7^ and participates in sister chromatid cohesion^8–11^. In addition, AND-1/Ctf4 facilitates homologous recombination (HR) repair of replication lesions^12^ and of DSBs in G2/M^6^.

AND-1/Ctf4 is involved in DNA replication by interacting with DNA Polymerase α and the CMG helicase complex^13–16^, bridging the CMG helicase to DNA polymerase α^7,17,18^. Recent results in budding yeast indicate that Ctf4 is a trimer^18^ and functions as a hub to recruit different other factors to the replication fork^19^. Thus, AND-1 emerged as a critical regulator of DNA replication-associated processes, but the chromosome lesions incurred upon AND-1 deficiency remain poorly understood.

AND-1 contains several functional domains such as the WD40 repeats in the N-terminal domain, the SepB domain in the central region, and the high mobility group (HMG) box in the C-terminal region^20^. WD40 repeats form ring-like beta-propeller structures that mediate protein-protein interactions^21^. The SepB domain, named from the AND-1 *Aspergillus nidulans* homolog, SepB, is the most conserved region in AND-1^18,22^. On the other hand, its C-terminal HMG box, a domain that is involved in DNA binding^23^, is unique to vertebrate AND-1.

While *CTF4* is not an essential gene in budding yeast, *AND-1/CTF4* orthologs in fission yeast, *Aspergillus nidulans* and Drosophila are essential for proliferation^18,22,24,25^. In human cells, AND-1 depletion by siRNA slows down proliferation by delaying the progression from late S through the G2 phase^6^. However, *AND-1* gene knockout cells or *AND-1* conditional knockout mutants have not been reported in vertebrates, making it difficult to study the immediate consequences of AND-1 loss while avoiding secondary and incomplete effects associated with siRNA knockdown.

To model the events stemming from uncoupling of the replicative helicase from the replisome and the roles of AND-1 in this process, here we established an effective conditional system of AND-1 depletion in genetically amenable avian DT40 cells^26,27^. Our results indicate that AND-1 is essential for proliferation in vertebrate cells. A single S phase without AND-1 induces slower replication forks and long ssDNA regions at the replication fork junctions. These gaps are converted into DSBs in G2, triggering checkpoint activation and cell cycle arrest. Notably, both the ssDNA accumulation at replication forks and damage accumulation in G2, but not the fork slow-down, are reversed by treatment with mirin, an MRE11 nuclease inhibitor^28^. Domain analysis further revealed that the HMG domain is critical for robust AND-1 enrichment at replication forks and fast replication fork speed, but not for proliferation. Vice-versa, we find that the WD40 repeat domain does not affect fork speed, but it is essential for proliferation and for averting formation of resected forks and subsequent DNA damage. Thus, the replication fork speed and fork protection functions of AND-1 are separable and mediated by distinct domains. The results indicate that the function of AND-1/Ctf4 in protecting replication forks from nucleolytic processing is essential for proliferation and for the maintenance of genome integrity, and is largely mediated via its WD40 domain.

## Results

### AND-1 is essential for proliferation

**Fig. 1:**
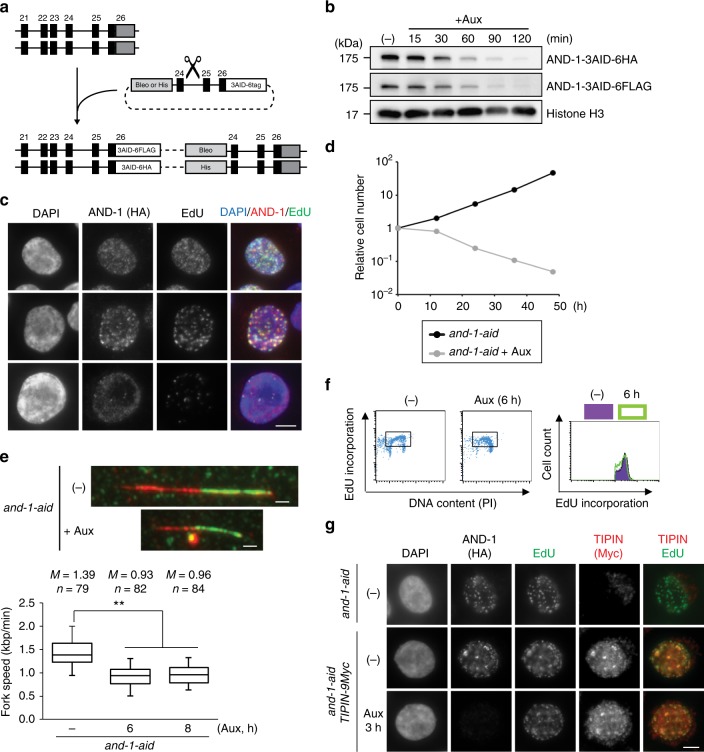
Establishment and characterization of AND-1 depletion in DT40 cell lines. **a** Schematic representation of the *AND-1* gene locus and flip-in constructs. Black boxes indicate exons, “Bleo” and “His” indicates drug resistance markers. **b** Total cell lysates were prepared from *and-1-aid* cells expressing AND-1-3-AID-6FLAG and AND-1-3AID-6HA, which were analyzed by Western blotting at the indicated time points. **c** *and-1-aid* cells were incubated with EdU for 15 min, and replication foci (marked by EdU) and AND-1 foci were visualized by Click-iT method and immunostaining with HA antibody, respectively. Scale bar represents 5 μm. **d** Growth curves of *and-1-aid* cells. 10^5^ cells were inoculated in 1 mL of medium and passaged every 12 h. **e** *and-1-aid* cells were cultured with or without auxin for indicated times, and DNA replication elongation rates were determined from the lengths of CldU tracks (only the ones clearly connected with IdU tracks were considered). Scale bar on the representative fiber image represents 5 kb. Middle line = median; box = 25th and 75th percentiles; bars = 5th and 95th percentiles. *M* indicates median values, *n*, the number of fibers analyzed in each condition. *P* values were calculated by Student’s *t*-test and **P ≤ 0.01. **f** Cell cycle distribution of AND-1 depleted cells. *and-1-aid* cells were incubated with auxin for 6 h, pulse-labeled with EdU for 15 min, and harvested. The cells were stained with Alexa488 azide to detect EdU uptake and with PI to detect DNA. Left panels show EdU uptake on the *y*-axis, and total DNA on the *x*-axis. Right panel shows cell number on the *y*-axis, and EdU uptake on the *x*-axis. **g** *and-1-aid* and *and-1-aid TIPIN-9Myc* cells were processed as in (**c**), with the addition of auxin treatment for 3 h, to monitor EdU, AND-1 (anti-HA), and Tipin (anti-Myc) foci. Scale bar represents 5 μm

To establish AND-1 conditionally depleted cells, we applied the auxin-inducible degron (AID) system, which enables rapid degradation of target proteins by the proteasome^27,29^ in chicken DT40 cell lines. We used a cell line that stably expresses *TIR1*, an essential component in the auxin degron system, from the β-actin promoter^30^. We C-terminally tagged the endogenous *AND-1* genes with the 3AID-6FLAG and 3AID-6HA tags, respectively, using the flip-in system for insertion of epitope tags^31^ (Fig. 1a). By sequential transfection of the epitope tags, we obtained *AND-1*^*3AID6FLAG/3AID6HA*^ cells expressing *TIR1* (thereafter referred to as *and-1-aid*) cells. The correct insertion of the AID tags to the carboxy termini of AND-1 was confirmed by western blotting (Fig. 1b). After auxin addition, both AND-1-3AID-6FLAG and AND-1-3AID-6HA proteins disappeared within 2 h (Fig. 1b). In the absence of auxin, *and-1-aid* cells behaved similarly with WT in regard to proliferation, replication fork speed and cell cycle distribution (Supplementary Fig. 1a-c). Next, to address if AID-tagged AND-1 protein is functional, we examined AND-1 recruitment to replication factories. We found that AND-1, visualized by anti-HA antibodies, co-localizes with replication foci marked by EdU (Fig. 1c).

We next examined the consequences of AND-1 depletion for cellular proliferation. *and-1-aid* cells stopped proliferating immediately after auxin addition (Fig. 1d). DNA fiber analysis revealed reduced DNA replication speed in AND-1 depleted cells (Fig. 1e), but intriguingly, overall replication monitored by EdU incorporation was not affected (Fig. 1f). This latter result was consistent with the one reported in human cells in which AND-1 levels were lowered by siRNA^6^.

We next addressed if replication fork slow-down in AND-1 depleted cells is caused by defective Tipin and/or Claspin localization to the fork, as these proteins interact with AND-1 and are required for fast replication fork speed^32–36^. To these ends, we tagged endogenous Tipin and Claspin (encoded by the *CLSPN* gene) C-terminally with 9Myc (Supplementary Fig. 2a) in *and-1-aid* cells, and examined their foci formation. Tipin and AND-1 foci co-localized with each other and with EdU foci, but AND-1 depletion did not impact on Tipin ability to form replication foci (Fig. 1g). Moreover, using chromatin fractionation, we found that the binding of Claspin to chromatin is not reduced upon AND-1 depletion (Supplementary Fig. 2b).

As overall replication monitored by EdU incorporation was not affected in the absence of AND-1 (Fig. 1f), we further checked if deregulated origin firing and/or fork collapse may diminish the inter-origin distance to support fast replication. In line with previous reports^37^, we found reduced inter-origin distance in *claspin* mutants, but no significant differences in AND-1 depleted cells (Supplementary Fig. 2c). Thus, AND-1 depleted cells have slow replication fork speed not associated with altered origin firing dynamics, and complete bulk replication before stopping their proliferation.

### AND-1 depletion causes DSBs and checkpoint activation in G2

**Fig. 2:**
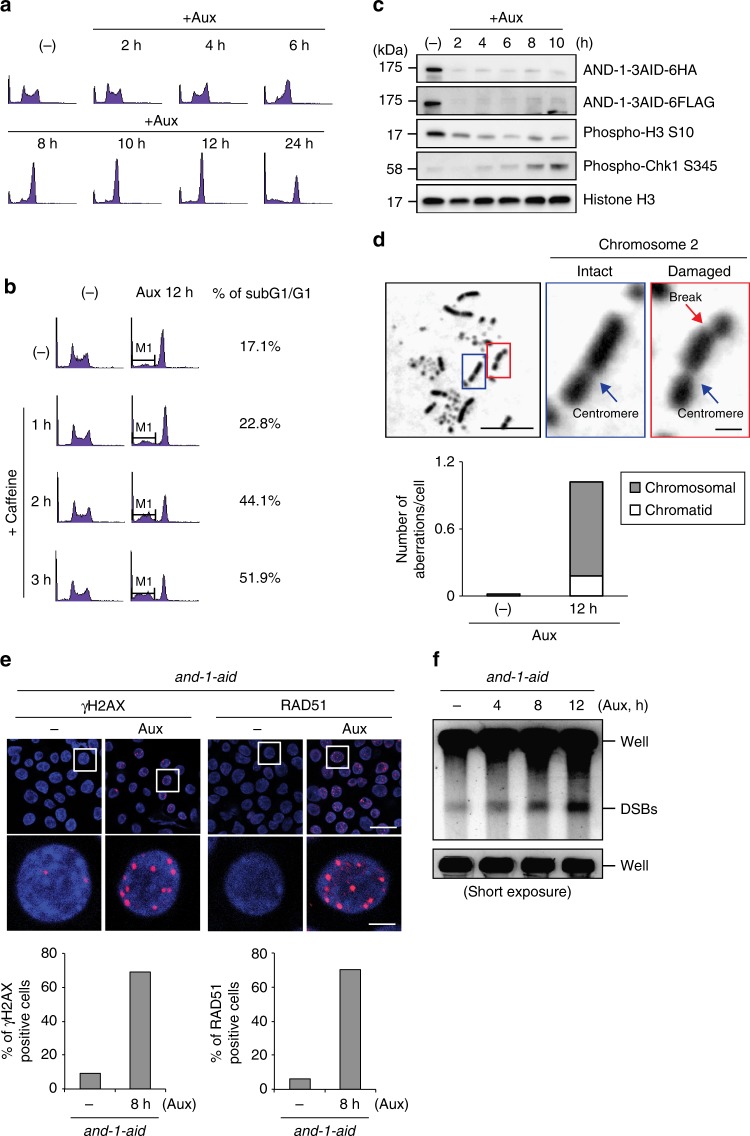
AND-1 depleted DT40 cells display spontaneous accumulation of DNA double strand breaks (DSBs) and DNA damage checkpoint activation. **a** Cell cycle distribution of AND-1 depleted cells. *and-1-aid* cells were incubated with auxin for indicated times, stained with propidium iodide (PI), and DNA content was analyzed by flow cytometry. **b** *and-1-aid* cells were incubated with or without auxin for 12 h, caffeine was added, and further incubated for the indicated times. DNA content was analyzed by flow cytometry. **c** Total cell lysates were prepared from *and-1-aid* cells at the indicated time points and analyzed by Western blotting. **d** *and-1-aid* cells were incubated with or without Auxin for 12 h, caffeine and colcemid were added, and further incubated for 2 h. Metaphase spreads were prepared from *and-1-aid* cells. Examples of intact and damaged chromosomes (the chromosomes are identified by shape in DT40) are shown. Scale bars represent 10 μm in left panel, 1 μm in enlarged picture (chromosome 2). **e** *and-1-aid* cells were incubated with auxin for 8 h and γH2AX or RAD51 foci were visualized by immunostaining with specific antibodies. Scale bars represent 25 μm in upper panels, 5 μm in enlarged pictures. **f** DSB detection via PFGE. *and-1-aid* cells were incubated with auxin for the indicated times, collected into agarose plugs and their DNA was separated by size on an agarose gel. Under the electrophoresis conditions used, high molecular weight genomic DNA remains in the well, whereas lower molecular weight DNA fragments (several Mb to 500 kb) migrate into the gel and are compacted into a single band

Since AND-1 depleted cells completed bulk replication with kinetics similar to control cells (Fig. 1f), yet stopped proliferating soon after auxin addition (Fig. 1d), we monitored their cell cycle distribution more precisely, harvesting cells every 2 h for cell cycle analysis. This experiment revealed that AND-1 depletion caused cells to gradually accumulate and subsequently arrest in G2/M (Fig. 2a). To address if the G2/M accumulation is the result of checkpoint activation and/or if AND-1 depleted cells fail cell division and stop during M phase, we added caffeine 12 h after auxin addition to inhibit the DNA damage checkpoint, as previously reported^38,39^. In the presence of caffeine, a large fraction of AND-1 depleted cells were released from the G2 arrest and proceeded to the next G1 phase, with concomitant increased accumulation of sub-G1 cells (Fig. 2b). Moreover, substantiating the notion that in AND-1 depleted cells spontaneously occurring DNA damage causes checkpoint activation and G2 arrest, we found that AND-1 depletion induces Chk1 phosphorylation (Fig. 2c). Chk1 phosphorylation could be detected in AND-1 depleted cells 4 h after auxin addition, with the signal maximizing 8–10 h after auxin addition when most AND-1 depleted cells were arrested in G2/M. We did not observe major effects on total Chk1 levels (Supplementary Fig. 3a).

To address the nature of the spontaneously arising DNA damage that causes checkpoint activation, we analyzed chromosome abnormalities of AND-1 depleted cells. To allow AND-1 depleted cells to reach metaphase, we first released them from the checkpoint-mediated G2 arrest using caffeine (see Fig. 2b), and then induced metaphase arrest by addition of colcemid. Notably, under these conditions, we observed a drastically increased number of chromatid and chromosome breaks, with the latter class being especially prominent (Fig. 2d).

In line with the notion that AND-1 depleted cells have increased levels of DNA damage, we detected γH2AX and RAD51 foci accumulation in these cells (Fig. 2e). Furthermore, pulse-field gel electrophoresis (PFGE) analysis revealed higher levels of broken DNA in AND-1 depleted cells (Fig. 2f). Taken together, the results indicate that in the absence of AND-1, cells can complete bulk DNA synthesis, but DSBs accumulate, triggering DNA damage checkpoint activation and subsequent G2 arrest.

### AND-1 loss causes long ssDNA stretches at replication forks

**Fig. 3:**
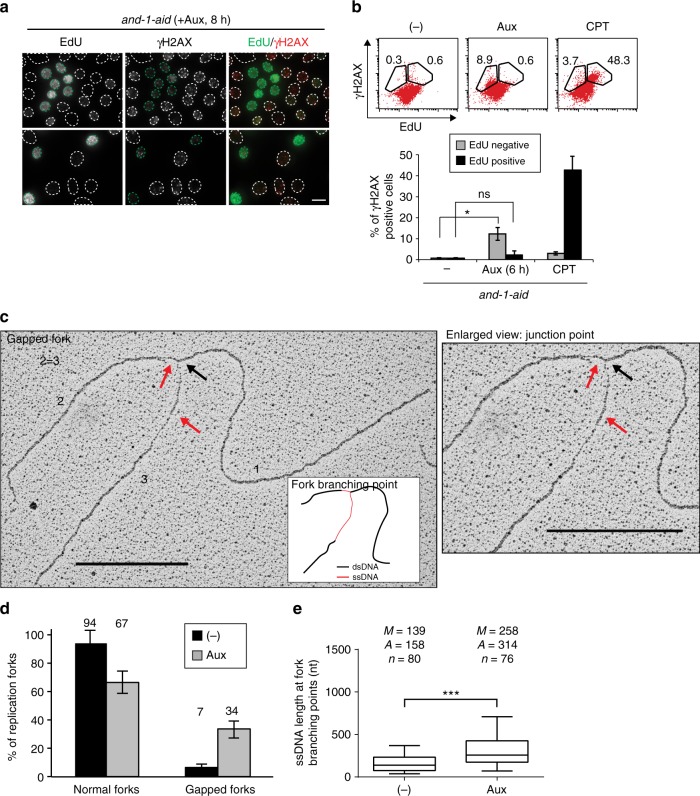
AND-1 depleted cells accumulate ssDNA at replication forks and DSBs in G2. **a**, **b** *and-1-aid* cells were cultured with auxin for 8 h, and incubated with EdU for 15 min. In case of CPT treatment, 10 μM CPT was added for 6 h after EdU incorporation and cells were incubated for 5 min. Cells in S phase or with DSBs were detected by Click-iT method or immnostaining with anti-γH2AX antibody. **a** Scale bar represents 10 μm. **b** Error bars represent standard deviation (SD) from three independent experiments. **c**–**e** *and-1-aid* cells were incubated with or without auxin for 4 h. Replication intermediates (RIs) were analyzed by transmission electron microscopy (TEM). **c** Representative TEM pictures (complete view and magnified image of the fork junction point) of a DNA replication fork with a single strand DNA discontinuity. The numbers 1, 2, 3 mark the three arms of the replication fork, with two arms, 2 and 3, being equal in length, using a 10% tolerance in measurements. For each TEM picture, 360 nm scale bars, corresponding to the length of 1 kb of DNA is shown. Red arrows point to the ssDNA filament, the black arrow to the fork branching point. A schematic representation of the junction point is shown, with dsDNA in black and ssDNA in red. **d** Chart and numbers above showing the percentages of replication forks analyzed in the indicated genetic backgrounds (the reported gapped forks have ssDNA > 300 nt). Molecules derive from two independent experiments, for a total number (*n*) of molecules shown in panel **e**. Error bars represent the SD obtained from two independent experiments. **e** Box plot reporting the distribution of ssDNA length at fork branching points. *n* is the total number of replication forks analyzed, *A* is the average length of ssDNA, and *M* is the median length of ssDNA. In the box plots, the middle line indicates the median value; the box shows the 25th and 75th percentiles; the bars, the 5th and 95th percentiles. *P* values were calculated by Student’s *t*-test. *** *P* ≤ 0.001

To address the timing of DSB formation versus active replication, AND-1 depleted cells were co-stained with γH2AX and EdU. Interestingly, most replicating cells marked by EdU staining (shown by white arrow) did not have γH2AX foci (Fig. 3a). To address the mutually exclusive staining patterns of γH2AX and EdU quantitatively, we employed FACS and used camptothecin (CPT)-treated cells as control, as it was reported that H2AX phosphorylation in response to CPT treatment occurs predominantly in S phase^40^. Untreated, auxin-treated, or CPT-treated *and-1-aid* cells were co-stained with γH2AX antibody, EdU and propidium iodide (PI). From γH2AX and PI staining, we confirmed that CPT-treated cells display strong H2AX phosphorylation predominantly in S phase, and found that auxin-treated *and-1-aid* cells displayed strong phosphorylation predominantly in G2/M (Supplementary Fig. 3b). Moreover, in line with this result, the γH2AX/EdU staining revealed that CPT treatment induced H2AX phosphorylation specifically in EdU positive cells, whereas conversely, AND-1 depletion induced γH2AX in EdU negative cells (Fig. 3b). To further address the observed inverse correlation between ongoing replication and γH2AX foci, we incubated cells with auxin and low concentration of HU. In this condition, the number of γH2AX and RAD51 foci observed upon AND-1 depletion was greatly reduced (Supplementary Fig. 3c), probably as a consequence of slowing down the replication speed and enriching cells in S phase (Supplementary Fig. 3d).

The nature of the replication fork-related DNA lesions, if any, induced by AND-1 depletion during normal replication are not known. Here, we used in vivo psoralen cross-linking and transmission electron microscopy (TEM)^41^ to visualize DNA replication intermediates generated after a single S phase without AND-1. We observed increased percentage of replication forks having abnormally long single stranded (ss) DNA at the fork branching point (gapped forks) in auxin-treated *and-1-aid* cells compared to untreated *and-1-aid* controls (Fig. 3c, d). Specifically, while 94% of the forks were normal, with no or relatively short stretches of ssDNA at the fork junctions in control *and-1-aid* cells, addition of auxin caused a drop in the normal forks and the appearance of 34% gapped forks. Gapped forks in AND-1 depleted cells were characterized by the presence of a long ssDNA discontinuity with an average length of 300 nt on one of the two replicated strands connected to the fork branching point (Fig. 3c–e). This asymmetric ssDNA discontinuity at the fork branching point was much shorter, with an average length of 150 nt, in the forks of the control cells (Fig. 3e). Taken together, the results indicate that the chromosome breaks observed in AND-1 depleted cells occur in the G2 phase and may originate from the long ssDNA stretches formed during DNA replication.

### AND-1 prevents nucleolytic processing of replication forks

The accumulation of gapped forks observed upon AND-1 depletion may imply a role for AND-1 in preventing fork resection, either by itself or via its ability to interact with replication fork protection factors that inhibit nucleolytic processing of the fork. As ssDNA gaps at the fork junction in AND-1 depleted cells correlate with increased RAD51 and γH2AX foci in G2, we reasoned that these foci may form as a consequence of the ssDNA accumulation at the fork junction, possibly as a result of nucleolytic processing of the fork. We asked if treatment of cells with mirin, an inhibitor of MRE11 previously implicated in fork resection^42–44^, could rescue RAD51 and γH2AX focus formation in AND-1 depleted cells. When asynchronous *and-1-aid* cells were treated with auxin and mirin for 8 h, we found that both RAD51 and γH2AX foci were reduced upon treatment with mirin (Supplementary Fig. 4a). However, mirin treatment also caused cells to be more enriched in S phase under these conditions (Supplementary Fig. 4b), and this S phase enrichment could potentially explain the reduction in the RAD51 and γH2AX foci (see Fig. 3a, b and Supplementary Fig. 3c-d). Release of *and-1-aid* cells from nocodazole arrest in the presence or absence of mirin, indicated that mirin addition does not affect the release, but delays S-phase entry and possibly S phase progression (Supplementary Fig. 4c).

**Fig. 4:**
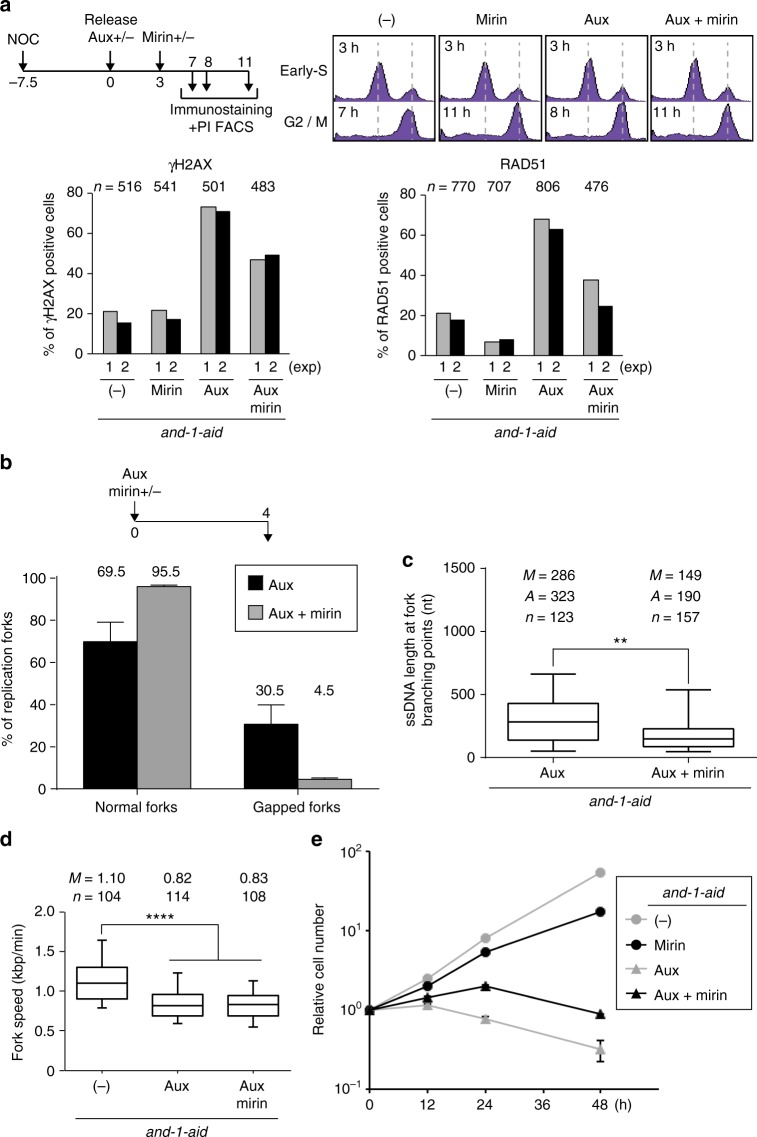
AND-1 prevents nucleolytic processing of the forks and subsequent damage accumulation. **a** Foci assay of γH2AX and RAD51. Upper panels: scheme of the experiment and cell cycle distribution at the indicated time points, when mirin was added and samples were collected for PI FACS and immunostaining. Cells untreated or treated with either or both mirin and auxin at indicated time points were stained with propidium iodide (PI), and DNA content was analyzed by flow cytometry. Bottom panels: γH2AX and RAD51 foci in G2/M cells untreated or treated with mirin, auxin, or both. Results of two experiments are shown. *n* represents the number of cells analyzed in the two experiments. **b**, **c** EM analysis of the replication intermediates purified from *and-1-aid* cells treated with auxin with or without mirin for 4 h as in Fig. 3d, e. Percentage or normal and gapped forks (**b**) and distribution of ssDNA length at the fork branching point (**c**). The reported gapped forks have ssDNA > 300 nt. Molecules derive from two independent experiments, for a total number (*n*) of molecules shown in **c**. *n* is the total number of replication forks analyzed, *A* is the average length of ssDNA, and *M* is the median length of ssDNA. In the box plots in **c**, the middle line indicates the median value; the box shows the 25th and 75th percentiles; the bars, the 5th and 95th percentiles. *P* values were calculated by Student’s *t*-test. ** *P* ≤ 0.01. **d** Fork speed measured as in Fig. 1e for and-1-AID cells untreated or treated with auxin, or auxin and mirin for 4 h as indicated in the scheme in Fig. 4b. ****P ≤ 0.0001. **e** Growth curves of *and-1-aid* cells in the indicated conditions. 10^5^ cells were inoculated in 1 mL of medium and passaged every 24 h. Error bars represent SD obtained from three independent experiments

To overcome mirin-induced S phase delays, we used a different experimental set-up in which cells reach G2/M in response to different treatments. Specifically, cells were arrested with nocodazole for 7.5 h, then released into medium with or without auxin. Mirin was added 3 h after release to half of the cultures, and samples were collected at different time points afterwards for PI FACS (Fig. 4a). The time points labeled as G2/M in the PI FACS analysis for the different conditions, namely, untreated (7 h), auxin (8 h), mirin (11 h), auxin plus mirin (11 h), were further processed for focus formation analysis. Also in this new set up, mirin treatment significantly reduced the accumulation of RAD51 and γH2AX foci in AND-1 depleted cells (Fig. 4a).

To address if the effects of mirin originate at the fork, we used TEM to examine the replication fork structure in AND-1 depleted cells treated or not with mirin (Fig. 4b, experimental scheme). Strikingly, addition of mirin strongly reduced the percentage of gapped replication forks in AND-1 depleted cells, from 32% observed in AND-1 depleted cells to 4% in AND-1 depleted cells additionally treated with mirin (Fig. 4b), and significantly reduced the average length of ssDNA gaps at the fork junction (Fig. 4c). Notably, mirin did not rescue the replication for speed delay characteristic of the AND-1 depleted cells (Fig. 4d), indicating that the functions of AND-1 in fork speed and fork protection are uncoupled. Moreover, addition of mirin, although toxic by itself, partly alleviated the proliferation defect in AND-1 depleted cells (Fig. 4e), and increased the percentage of cells that recover from auxin-induced G2 arrest (Supplementary Fig. 4d). Altogether, these results uncover a fork protection function of AND-1 that is critical in preventing fork resection and subsequent damage-induced lethality.

### AND-1 facilitates fast fork speed via its HMG domain

**Fig. 5:**
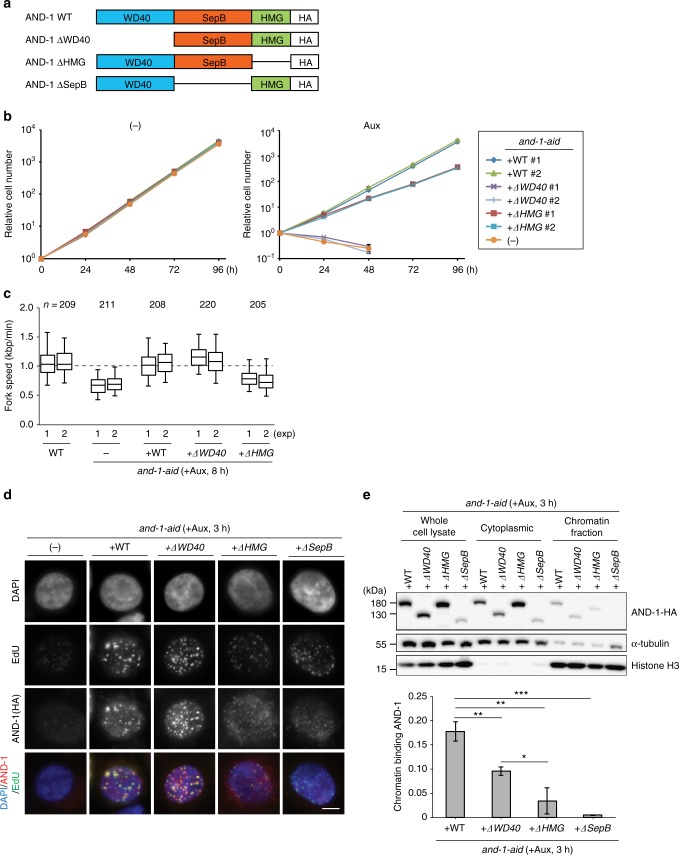
Identification of critical AND-1 domains for DNA replication and proliferation. **a** Schematic presentation of the generated AND-1 deletion variants. **b** Growth curves of cell lines of the indicated genotype. A total of 10^5^ cells were inoculated in 1 mL of medium and passaged every 24 h. Error bars represent SD obtained from three independent experiments. **c** DNA replication elongation rates were determined as shown in Fig. 1e, with *n* representing the numbers of fibers analyzed in each condition. **d** Cells of the indicated genotypes were cultured in the presence of auxin for 3 h and incubated with EdU, and replication foci and AND-1 foci were visualized by Click-iT method and immunostaining with HA antibody. Scale bar represents 5 μm. **e** Ratio of AND-1-HA and histone H3 in chromatin fractionation. Cells of the indicated genotypes were cultured in the presence of auxin for 3 h. Chromatin bound AND-1 and histone H3 were assessed by immunoblotting. Measured chromatin bound AND-1-HA amount normalized to histone H3 in the cell lysate of three independent experiments were averaged and plotted. Error bars represent standard error of the mean (SEM) obtained from three independent experiments. *P* values were calculated by Student’s *t*-test. *P represents *P* values smaller than 0.05, **P values smaller than 0.01, and ***P values smaller than 0.001

AND-1 has several distinguishable domains including WD40 repeats, SepB and HMG. The roles of its different domains in binding and stimulating the helicase activity of the human CMG complex has been in part addressed^15^. AND-1 binding to polymerase α is mediated by its SepB domain^18^; in addition, a contribution for the HMG domain was proposed in mammalian cells^16^. However, the critical domains of AND-1 in DNA replication, cell survival or its localization remain unknown. To address the latter questions, we amplified full-length chicken *AND-1* cDNA containing the HA tag by RT-PCR and cloned it to an expression vector in which *AND-1-HA* is expressed from the chicken β-actin promoter. From the full-length *AND-1-HA*, we made ΔSepB, ΔWD40 and ΔHMG mutants, and transfected them to *and-1-aid* cells (Fig. 5a). As SepB is the most highly conserved domain of AND-1 that binds to Polymerase α and Sld5, we first analyzed the *and-1-*ΔSepB mutant. As expected based on its high conservation^18^, this domain was essential for all AND-1 functions, judging from the fact that the growth defects (Supplementary Fig. 5a), replication fork slowing (Supplementary Fig. 5b), accumulation of spontaneous γH2AX and RAD51 foci (Supplementary Fig. 5c-d), cell cycle arrest (Supplementary Fig. 5e) characteristic of AND-1 depleted cells failed to be suppressed by the expression of the AND-1 ΔSepB variant, whereas all these phenotypes were suppressed by the expression of AND-1 WT (see below).

Next, we analyzed *and-1* ΔWD40 and ΔHMG mutants. The growth curve of these mutants revealed that the WD40 domain, but not the HMG box, is essential for proliferation, although proliferation also becomes slower in the absence of the HMG box (Fig. 5b). Conversely, when we monitored the replication fork speed of these mutants, we found that *and-1-ΔHMG*, but not *and-1-ΔWD40*, had shorter DNA replication tracts (Fig. 5c). It is reasonable to assume that AND-1 firmly binds to DNA via its HMG box, since the HMG domain is known as a DNA binding motif^23^. Thus, our results suggest that reduced DNA binding of AND-1 causes replication fork slowing and results in mild proliferation defects.

To address this hypothesis further, we examined the capability of AND-1 protein and variants to engage in replication factories. We found that AND-1 focus formation was reduced in the *and-1-ΔHMG* and *and-1-ΔSepB* mutants, but not in the *and-1-ΔWD40* mutant (Fig. 5d). The same trend was observed via chromatin fractionation assay, in which we found reduced chromatin binding for all AND-1 mutant variants, but stronger reduction was observed with the ΔΗΜG mutant compared to the ΔWD40 variant. Very little binding was observed for the ΔSepB variant, although in this case, the expression levels were lower to begin with, likely due to reduced protein stability in the absence of the SepB domain, making the direct comparison with the other variants difficult (Fig. 5e).

To investigate further the role of the HMG domain of AND-1 in replication fork speed and to compare it with those caused by mutations in Claspin and Tipin, we constructed *and-1* knockout cell lines expressing the ΔΗΜG variant (Supplementary Fig. 6a-b). In this case there is no residual AND-1 protein caused by incomplete AND-1 depletion. The *and-1* knockout cells expressing the ΔΗΜG variant cells showed reduced replication fork speed, but the defects were modest in comparison with those of *tipin* and *claspin* mutants (Supplementary Fig. 6c). Similarly, the proliferation defects of ΔΗΜG cells were milder than those of *tipin* and *claspin* (Supplementary Fig. 6d) and showed similar trends with those of AND-1 depleted cells expressing the ΔΗΜG variant (see Fig. 5b).

These results suggest that robust binding of AND-1 to DNA mediated by its HMG box is required for fast replication fork speed, but it is not essential for proliferation, likely because the basic and essential amount of AND-1 on chromatin is supported by SepB- and WD40-mediated protein interactions.

### AND-1 WD40 repeats prevent resected forks and DSBs in G2

**Fig. 6:**
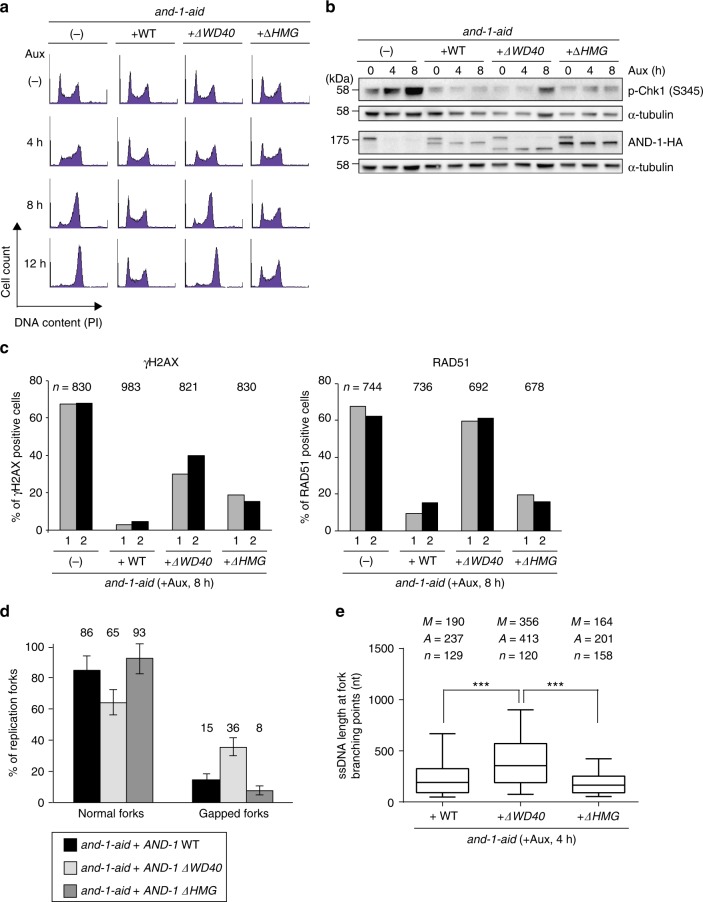
Identification of AND-1 domains critical for proliferation and prevention of spontaneous DSB formation. **a** Cell cycle distribution of AND-1 deletion mutants. Cells of the indicated genotypes were incubated with auxin for indicated times, stained with propidium iodide (PI), and DNA content was analyzed by flow cytometry. **b** Total cell lysates were prepared from cells of the indicated genotypes and analyzed by Western blotting. **c** Cells of the indicated genotypes were incubated with auxin for 8 h and γH2AX and RAD51 foci were visualized by immunostaining with specific antibodies. Results of two experiments are shown. *n* represents the number of cells analyzed in the two experiments. **d, e** EM analysis of the replication intermediates purified from the cell lines of the indicated genotype as in Fig. 3d, e. Molecules derive from two independent experiments, for a total number (*n*) of molecules shown in **e**. *n* is the total number of replication forks analyzed, *A* is the average length of ssDNA, and *M* is the median length of ssDNA. Error bars in **d** represent SDM obtained from two independent experiments. In the box plots in **e**, the middle line indicates the median value; the box shows the 25th and 75th percentiles; the bars, the 5th and 95th percentiles. *P* values were calculated by Student’s *t*-test. ***P  ≤ 0.001

Next, we monitored cell cycle distribution, checkpoint activation, and γH2AX focus formation in *and-1-aid* mutants. Similar to cells depleted for AND-1, *and-1-ΔWD40*, but not *and-1-ΔHMG* cells gradually accumulated in the G2 phase after auxin addition (Fig. 6a). and-1-*ΔWD40* mutants also showed spontaneous Chk1 phosphorylation, although to a reduced extent compared to AND-1 depleted cells (Fig. 6b). Spontaneous formation of γH2AX foci was also more frequently observed in *and-1-ΔWD40* than in *and-1-ΔHMG* mutants, although both variants had higher levels of γH2AX than WT AND-1 complemented *and-1-aid* cells (Fig. 6c). Notably, RAD51 foci were specifically increased in *and-1-ΔWD40* cells, and not in *and-1-ΔHMG*, to similar levels as in AND-1 depleted cells (Fig. 6c). These results indicate that AND-1 roles in averting DSB formation in G2 and subsequent checkpoint activation-mediated cell cycle arrest are primarily mediated by the WD40 repeats and its SepB domain.

The phenotype of G2 arrest in AND-1 depleted cells correlates with accumulation of ssDNA stretches proximal to replication fork branching points. The ssDNA accumulation proximal to replication fork junctions may be directly related with replication fork speed, which is reduced in AND-1 depleted and *and-1-ΔHMG* cells, or rather represent chromosome lesions caused by loss of AND-1 and subsequently triggering DSB formation and checkpoint activation. The AND-1 variants generated here with specific defects either for replication fork speed or for proliferation, offered the possibility to test these hypotheses. When we compared the length of ssDNA at replication forks and the percentage of normal versus gapped forks in different *and-1* mutants, we specifically observed long ssDNA at replication forks in the *and-1-ΔWD40* mutant, but not in the *and-1-ΔHMG* mutant or *and-1-aid* cells complemented with WT *AND-1* (Fig. 6d, e). Thus, the WD40 domain, and not the HMG domain necessary for physiologically fast levels of DNA replication and robust enrichment of AND-1 in replication factories, is essential to prevent ssDNA formation at replication fork junctions.

Considering that mirin treatment could also significantly reduce gapped fork formation (Fig. 4b) and RAD51 focus accumulation in G2 (Fig. 4a), the results imply that the fork protection function of AND-1 against nucleolytic cleavage by MRE11 is largely mediated via its WD40 domain. As RAD51 foci are increased in AND-1 depleted cells and in *and-1-ΔWD40* mutants, and reduced by mirin treatment, these results suggest that RAD51 is recruited to the ssDNA gaps at the fork junction after these are formed via MRE11-mediated nucleolytic processing. This could explain why AND-1 depleted cells are not protected by RAD51 against MRE11-mediated cleavage^44^. Moreover, the results indicate that the DSBs formed in AND-1 depleted cells derive in part from long ssDNA formed proximal to the replication fork junction as both are prevented by the WD40 repeat domain of AND-1 and by treating cells with mirin.

## Discussion

AND-1/Ctf4 is a critical component of the replisome, bridging polymerase α with the replicative CMG complex, and acting as a scaffold for various proteins that are recruited to the replication fork^7,17–19^. Intriguingly, although the bridging function of Ctf4/AND-1 is common in all the organisms studied to date, and likely shared with MCM10^45^, its requirement for proliferation function is not^5,22,24,25^.

Here we set out to identify the type of replication stress caused by defective AND-1-mediated bridging between the CMG helicase and the polymerase α and how this impacts proliferation in vertebrate cells. To these ends, we established *and-1-aid* conditional mutants in genetically amenable chicken DT40 cells. This system allows fast and complete degradation of AND-1, permitting analysis and dissection of the phenotypes induced by its loss.

We uncovered that cells can complete bulk DNA replication without AND-1, but newly synthesized DNA presents long ssDNA gaps proximal to the replication fork junction. We present evidence that lethal DSBs that activate the DNA damage checkpoint and stall cell cycle progression arise after completion of DNA replication and most likely originate from the ssDNA stretches formed during replication. This conclusion is not only correlative, based on the timing of the observed phenotypes—ssDNA first, at the replication fork junction, and then DSBs after the bulk replication is complete—, but also it has a genetic basis as AND-1 variants defective in suppressing the ssDNA accumulation at replication forks are also specifically defective in proliferation and show prominent G2 arrest. Moreover, addition of mirin, which inhibits the MRE11 nuclease activity, reverses not only ssDNA gap accumulation at the fork junction, but also the formation of DNA damage foci and prominent G2 arrest in AND-1 depleted cells. Thus, prevention of persistent ssDNA accumulation during replication underpins the critical function of AND-1 in proliferation.

Interestingly, yeast Ctf4 is also important to prevent ssDNA accumulation at replication forks in the presence of DNA damage and is required for viability under damaging conditions^12^. Thus, it seems that AND-1/Ctf4 has evolutionally conserved roles in averting ssDNA accumulation at replication forks. We posit that the different requirement for proliferation of vertebrate AND-1 and budding yeast Ctf4 is related to the frequency of the replication-related events causing ssDNA formation, with this being much higher in vertebrate cells. An implication of our findings is that a common function of AND-1/Ctf4 conserved across organisms is related to the prevention and effective management of those gapped ssDNA containing regions, often dealt with by subsequent recombination events^12^.

Our work reveals that in vertebrate cells lacking AND-1 the gapped forks arise in large part from unscheduled nucleolytic events, thus identifying a role for AND-1 in replication fork protection. The findings that RAD51 is bound to chromatin in AND-1 depleted and *and-1-ΔWD40* mutants, but does not protect against nucleolytic cleavage, suggest that RAD51 is recruited after ssDNA gap formation took place. Alternatively, it is possible that the mode of binding of RAD51 in AND-1 depleted cells is defective and does not offer the same level of fork protection^44^, as is the case with certain RAD51 variants^46^. This latter hypothesis may explain the subsequent formation of DSBs, which cause prominent cell cycle arrest in G2, likely due to improper repair.

Importantly, our results indicate that the WD40 repeat domain of AND-1, and not the HMG box, is essential in preventing ssDNA and DSB accumulation coupled with cell cycle arrest. In many organisms, replication fork restart and protection strongly rely on HR factors and mediators^42–44^. Notably, DSB accumulation in G2/M has also been observed upon depletion or inactivation of HR factors in vertebrate and mammalian cell systems^39,47,48^, and critical interactors of the WD40 domain of the PALB2 recombination protein involves binding to other HR factors, such as RAD51C, RAD51, and BRCA2^49^. Although RAD51 binding appears proficient in AND-1 depleted cells, our results indicate that this binding is not sufficient to prevent nucleolytic processing of the forks in cells lacking AND-1 and is also not sufficient to promote repair in G2^39^. We thus propose that AND-1/Ctf4 essential function, executed in large part via its WD40 repeats, is to provide fork protection, and thereby to promote genome integrity.

## Methods

### Cell lines

The cell lines and the targeting constructs are described in the Supplementary Table 1.

### Cell culture

Cells were cultured at 39.5 °C in D-MEM/F-12 medium (Gibco) supplemented with 10% fetal bovine serum, 2% chicken serum (Sigma), penicillin/streptomycin mix, and 10 μM 2-mercaptoethanol (Gibco) in the presence or absence of 500 μM auxin. To plot growth curves, each cell line was cultured in three different wells of 24 well-plates and passaged every 12 h or 24 h. Cell number was determined by flow cytometry using plastic microbeads (07313-5; Polysciences). Cell solutions were mixed with the plastic microbeads suspension at a ratio of 10:1, and viable cells determined by forward scatter and side scatter were counted when a given number of microbeads was detected by flow cytometry.

### Antibodies and Western blots

The antibodies used for immunoblotting were the ones against HA (1:2000, 11867423001, Roche), Myc (1:2000, clone 9E10, produced by IFOM facility), Chk1 (sc8408, Santa Cruz), Chk1-P (1:1000, 2341, Cell Signaling Technology), RPA (1:1000, A300-244A, Bethyl), Histone H3 (1:2000, ab1791, Abcam), Histone H3-P (1:1000, 06-570, Millipore), α-tubulin (1:3000, T5168, Sigma) and FLAG (1:2500, F3165, Sigma).

All uncropped blots in the main figures are shown in Supplementary Fig. 7.

### Plasmid construction and transfection

To add 3xmAID-6xFLAG and 3xmAID-6xHA tags to AND-1 by Flip-in system, 2.3 kb upstream DNA sequences of stop codons of *AND-1* was amplified with the primers 5′- TTTTGTCGACGTTGTTTGAGGGTGGCACTGCTGC-3′ (SalI) and 5′- TTTTACTAGTGCTCTGCTTGAATGCAAAAGAGGAC-3′ (SpeI). The amplified DNA fragment was cloned into p3xmAID-6xFLAG or p3xmAID-6xHA vector at SalI and NheI restriction enzyme sites^30^. The Flip-in vectors were then linearized at one restriction enzyme site in the middle of the homology region and transfected to DT40 cells as previously described^31^.

For AND-1 cDNA cloning, we used primers 5′- TTTCGGCCGGATATCATGCCGTCAGAGCAAAAGCCGATGC -3′ (EagI + EcoRV) and 5′- TTTGCGGCCGCTTAAGCGTAGTCTGGGACGTCGTATGGGTAGCTCTGCTTGAATGCAAAAGAGGAC -3′ (NotI + HA). The amplified DNA fragment was cloned into pTRE2-hygro vector (Clontech). The promoter was replaced by chicken beta-actin promoter and the hygromycin-resistant marker was replaced by the puromycin-resistant marker.

To make AND-1 deletion mutants (AND-1 ΔSepB, ΔWD40 and ΔHMG), we used WT *AND-1-HA* cDNA as a template and removed corresponding DNA sequences using the primers 5′- TACTGCCAAGTTACAACAGAAAAGGGGCAG -3′ and 5′- CTTTTGCCTGGGGGTTGGCCTTGGCCCATC -3′ (for ΔSepB); and 5′- TATACAGATACTGAAGGAAATCTGGGATTG -3′ and 5′- CCGCATCGGCTTTTGCTCTGACGG -3′ (for ΔWD40); and 5′- AAGCGTCCCACAGCTGCTGAAGATGAG-3′ and 5′- GTTTTCAGTGTTGTCCTGTGCAGTGAC-3′ (for ΔHMG).

To add 9xMyc tags to Claspin and Tipin by flip-in system, 2.3 kb upstream DNA sequences of stop codons of *CLSPN* and 2.2 kb upstream DNA sequences of stop codons of *TIPIN* were amplified with the primers 5′- AAAAGTCGACGTGGGCAGTGAGGATGAGTACG -3′ (SalI) and 5′- AAAACCTAGGGCTCTCCAAGTACTGGAATATGCTTC -3′ (AvrII) for *Claspin* and 5′- AAAAGTCGACTGACAGCTGTAAAAACCAAACTCAG -3′ (SalI) and 5′- AAAACCTAGGCTGCTTTTCACTGGCACACTGCAATTC -3′ (AvrII) for *Tipin*. The amplified DNA fragments were cloned into p9xMyc vector at SalI and NheI restriction enzyme sites^50^. The flip-in vectors were then linearized at one restriction enzyme site in the middle of the homology region and transfected to DT40 cells as previously described^31^.

*AND-1* KO-Bsr and *AND-1* KO-Bleo were generated from genomic PCR products combined with blasticidin S and bleomycin selection marker cassettes. Genomic DNA sequences were amplified using primers 5′- Gttgtcgactgcctgcttgcttctctgttg (SalI) -3′ and 5′- Tttggtaccacaagataacagcccatagcccag (KpnI) -3′ (for the right arm of the KO construct); and 5′- gttactagtgcagcacatttcagcctgtttg (SpeI) -3′ and 5′- gttactagtatgcccaaggagccaaagaactg (SpeI) -3′ (for the left arm of the KO construct). Amplified PCR products were purified by gel extraction and cloned into pLoxP vectors by digesting with SalI-HF and KpnI for the right arm, SpeI for the left arm. Briefly, 152 bp of the coding sequence of *AND-1* was replaced by drug resistance marker cassettes, causing deletion of 64–113 amino acids and a frameshift mutation. *AND-1* knockout vectors were then linearized with Not I restriction enzyme before transfected to DT40 cells.

### DNA fiber analysis

DNA fiber analysis was performed as previously described^32^ with small modifications. Cells (5 × 10^5^ in 1 mL of medium) were pulse-labeled with 25 μM chlorodeoxyuridine (CldU; Sigma) and then sequentially pulse-labeled with 250 μM iododeoxyuridine (IdU; Sigma). Cells were resuspended in ice-cold PBS and then daubed on glass slides. Cells were lysed with DNA fiber lysis buffer (0.5% SDS, 200 mM Tris-HCl, pH 7.4, 50 mM EDTA), and then glass slides were tilted to extend DNA. For fixation, glass slides were immersed in Carnoy fluid (MeOH:AcOH, 3:1) for 3 min, 70% EtOH for 1 h. After washing with PBS, glass slides were immersed in 2.5 M HCl for 30 min to denature DNA molecules and subsequently in 0.1 M sodium tetraborate for 3 min to neutralize. After washing with PBS, the slides were treated with rat anti-BrdU antibody (1:200; Abcam) and mouse anti-BrdU antibody (1:50; BD Biosciences), which reacted against CldU and IdU, respectively. Cy3-conjugated anti-rat IgG (1:400; Jackson ImmunoResearch Laboratories) and Alexa Fluor 488 anti-mouse IgG (1:100; Invitrogen) were used as the secondary antibodies. The first and second antibodies were incubated for 1 h each at room temperature. Washing of antibodies was performed with 0.05% Tween 20 in PBS. Coverslips were mounted with PermaFluor mounting medium (Lab vision). Images were captured with a fluorescence microscope. Fiber lengths were measured using ImageJ. DNA replication elongation rates were calculated as CldU fiber length divided by pulse-labeling time.

### Detection of DSBs by pulse-field gel electrophoresis (PFGE)

PFGE analysis was performed as previously reported with small modifications^51^. Cells were harvested and washed with ice cold PBS once. Then cells were incubated with ice cold 0.075 M KCl for 5 min. After centrifugation, 0.7% agarose plugs containing 10^6^ cells were prepared with a CHEF-disposable plug mold (Bio-Rad). The cells were lysed by incubation of the plugs in 1 mg/ml proteinase K in 0.5 M EDTA, 1% N-Lauroylsarcosine, 10 mM Tris-HCl (pH 8.0) for 24 h at 50 °C and then washed once with 10 mM Tris–HCl (pH 8.0), 100 mM ETDA. Electrophoresis was performed for 21 h at 13 °C through 0.9% agarose in Tris–borate–EDTA buffer using a CHEF-DR III apparatus with the following parameters: interval, 30–18 s; angle, 120° 5.5 V/cm for 9 h: interval, 18 to 9 s; angle, 117° 4.5 V/cm for 6 h and interval, 9 to 5 s; angle, 112° 4.0 V/cm for 6 h. The DNA was stained with ethidium bromide and visualized using a FAS-IV (Nippon genetics). The electrophoresis conditions were specifically designed to compact lower molecular weight DNA fragments (several Mbp to 500 kbp) into a single band, while keeping high molecular weight genomic DNA in the well.

### Detection of EdU incorporation and γH2AX by FACS

Cells were cultured in the presence of EdU for 15 min, fixed in 70% ethanol, incubated with anti γH2AX antibody (Merck Millipore), and stained with Alexa647-conjugated anti-mouse IgG antibody (Thermo Fisher Scientific). After immunostaining, Click-iT reaction was performed with manufacturers’ instruction with Alexa488-conjugated Fluor® azide (Thermo Fisher Scientific). Finally, cells were stained with propidium iodide and analyzed by FACS.

### Electron microscopy

The in vivo psoralen cross-linking, the genomic DNA preparation was performed as previously described^41^, and the protocol was optimized for DT40 cells as described in the following. In brief, for the in vivo psoralen cross-linking cellular pellets corresponding to 0.5–1 × 10^7^ cells were washed and resuspended in 10 ml of ice cold PBS. Two cycles of 5 min each of in vivo psoralen cross-linking were performed by placing the cell suspensions (transferred in 6 cm dish for tissue culture) in a Stratagene Stratalinker 2400 irradiation chamber containing five bulbs of 15 watts each (Cat number 400079), with 365 nm emitting wave length (UV-A), for an estimated total dose of 1000 mJ/cm^2^. Before each irradiation cycle, a fresh 0.5-ml aliquot of psoralen (4, 5′, 8 trimethyl-psoralen (TMP) 0.2 mg/ml dissolved in ethanol) was added to the cell suspension, mixed with cut micropipette tips and incubated for 5 min in the dark. During the entire procedure, the plates containing the cell suspensions were placed on ice. During the irradiation cycles, the cell suspension was 3 cm distant from the bulbs. After this procedure, the cell suspensions were transferred to 50-ml conical tubes, the pellets were washed twice with ice cold PBS, and genomic DNA was extracted as previously reported^41^. DNA was partially digested by PvuII-HF and the DNA fragments containing ssDNA were enriched by a column filled with BND cellulose^41^. Enriched DNA samples were subjected to DNA spreading on carbon-coated metal grids (4-nm thickness) in the presence of uranyl acetate, and this was followed by platinum-based rotatory shadowing (0.4 nm without rotation and up to 8 nm with rotation)^41^. EM analysis was performed as previously described^41^.

### Data availability

All relevant data are available from the authors upon reasonable request.

## Electronic supplementary material

Supplementary Information
