# Supplementary material for: AND-1 fork protection function prevents fork resection and is essential for proliferation
Source: Nat Commun. 2018 Aug 6;9:3091. doi: 10.1038/s41467-018-05586-7 (PMC6079002; doi:10.1038/s41467-018-05586-7)
Supplement: Supplementary file 1 — Supplementary Information [file 41467_2018_5586_MOESM1_ESM.pdf]

AND-1 fork protection function prevents fork resection and is essential for proliferation

Abe, Kawasumi et al

Supplementary Table 1. Cell lines used in the study.

Genotype	Selective marker	Reference	Strain No.	
WT	-	(26)	8	
AND-1$^{3xmAID-6xFLAG/3xmAID-6xHA}$ +*TIR1*^{9xMyc}	*AND-1/AND-1*::3xmAID-6xHA FLP-In-His/3xmAID-6xFLAG FLP-In-Bleo, +*TIR1-9xMyc*::Bsr	This study	527	
AND-1$^{3xmAID-6xFLAG/3xmAID-6xHA}$ +*TIR1*^{9xMyc} +*AND-1*^{HA}	*AND-1/AND-1*::3xmAID-6xHA FLP-In-His/3xmAID-6xFLAG FLP-In-Bleo, +*TIR1-9xMyc*::Bsr, +*AND-1-HA*::Puro	This study	565	R205
AND-1$^{3xmAID-6xFLAG/3xmAID-6xHA}$ +*TIR1*^{9xMyc} +*AND-1*^{HA} *ΔSepB*	*AND-1/AND-1*::3xmAID-6xHA FLP-In-His/3xmAID-6xFLAG FLP-In-Bleo, +*TIR1-9xMyc*::Bsr, +*AND-1-HA ΔSepB*::Puro	This study	R209	R210
AND-1$^{3xmAID-6xFLAG/3xmAID-6xHA}$ +*TIR1*^{9xMyc} +*AND-1*^{HA} *ΔWD40*	*AND-1/AND-1*::3xmAID-6xHA FLP-In-His/3xmAID-6xFLAG FLP-In-Bleo, +*TIR1-9xMyc*::Bsr, +*AND-1-HA ΔWD40*::Puro	This study	567	R208
AND-1$^{3xmAID-6xFLAG/3xmAID-6xHA}$ +*TIR1*^{9xMyc} +*AND-1*^{HA} *ΔHMG*	*AND-1/AND-1*::3xmAID-6xHA FLP-In-His/3xmAID-6xFLAG FLP-In-Bleo, +*TIR1-9xMyc*::Bsr, +*AND-1-HA ΔHMG*::Puro	This study	569	570
AND-1$^{3xmAID-6xFLAG/3xmAID-6xHA}$ *CLASPN*$^{+/9xMyc}$ +*TIR1*^{9xMyc}	*AND-1/AND-1*::3xmAID-6xHA FLP-In-His/3xmAID-6xFLAG FLP-In-Bleo, *CLASPN/CLASPN*::-/9xMyc FLP-In-Eco,+*TIR1-9xMyc*::Bsr	This study	R212	
AND-1$^{3xmAID-6xFLAG/3xmAID-6xHA}$ *TIPIN*$^{+/9xMyc}$ +*TIR1*^{9xMyc}	*AND-1/AND-1*::3xmAID-6xHA FLP-In-His/3xmAID-6xFLAG FLP-In-Bleo, *TIPIN/TIPIN*::-/9xMyc FLP-In-Eco,+*TIR1-9xMyc*::Bsr	This study	R211	
AND-1$^{-/-}$ +*AND-1*^{HA} *ΔHMG*	*AND-1/AND-1*::KO-Bsr/KO-Bleo, +*AND-1-HA ΔHMG*::Puro	This study	584	
CLSPN$^{-/-}$ +*tetoff-hCLSPN*^{HA}	*CLSPN/CLSPN*::KO-His/KO-Eco, +*tetoff-hCLSPN-HA*::Hyg	(32)	45	
CLSPN$^{-/-}$	*CLSPN/CLSPN*::KO-His/KO-Eco	This study	R235	
TIPIN$^{-/-}$	*TIPIN/TIPIN*::KO-Puro/KO-Bsr	(34)	46	

Supplementary Figures

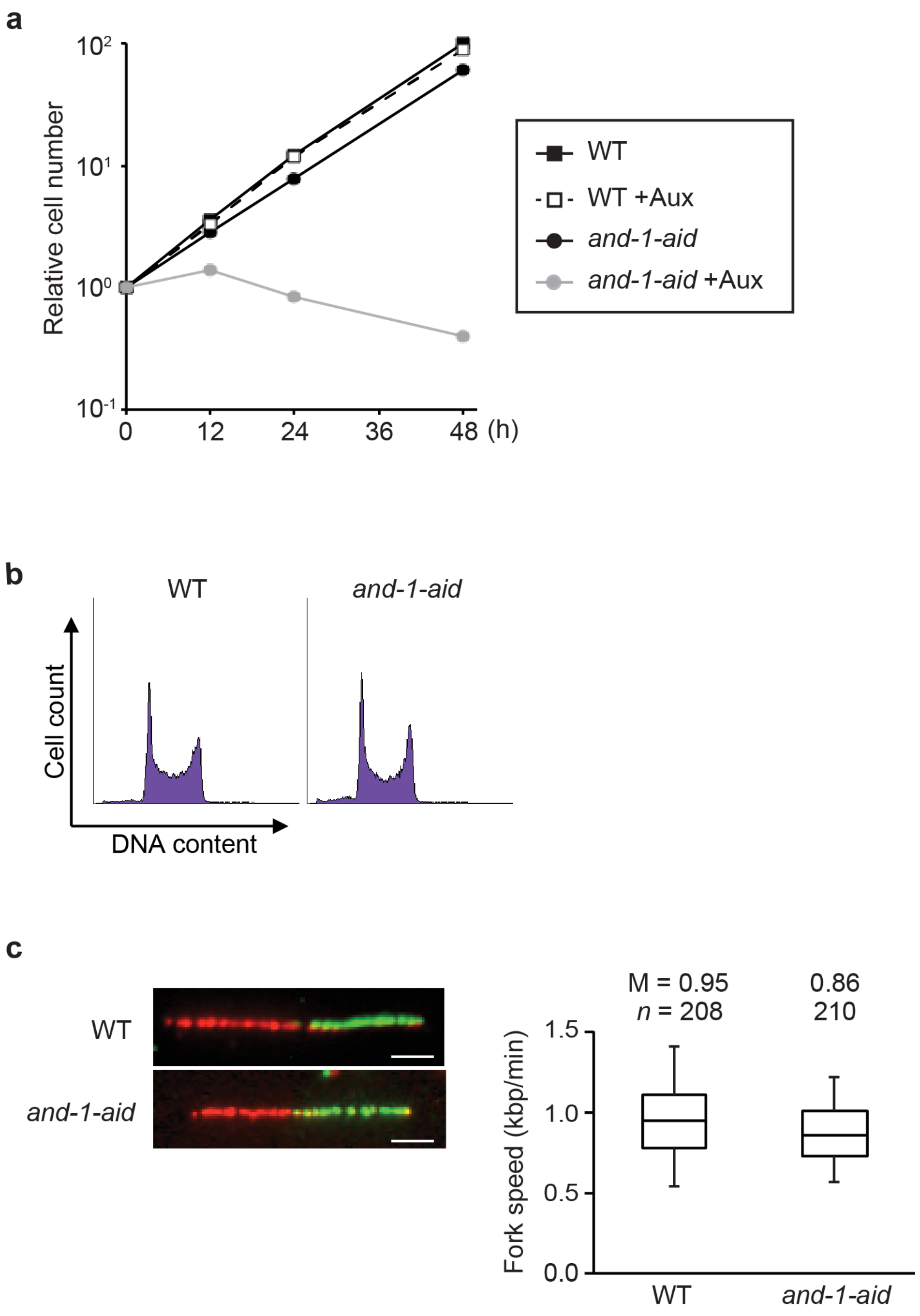

Supplementary Figure 1. Comparison between WT and *and-1-AID* cells.

a Growth curves of WT and *and-1-aid* cells. 10^5 cells were inoculated in 1 mL of medium and passaged every 12 h. **b** Cell cycle distribution of WT and *and-1-aid* cells.

Cells were stained with propidium iodide (PI), and DNA content was analyzed by flow cytometry. **c** DNA replication elongation rates of WT and *and-1-aid* cells, derived from the lengths of CldU tracks (only the ones clearly connected with IdU tracks were considered). Scale bar on representative image represent 5 kb. Middle line = median; box = 25th and 75th percentiles; bars = 5th and 95th percentiles. M indicates median values, n, the number of fibers analyzed in each condition. Similar trend was observed in an independent experiment.

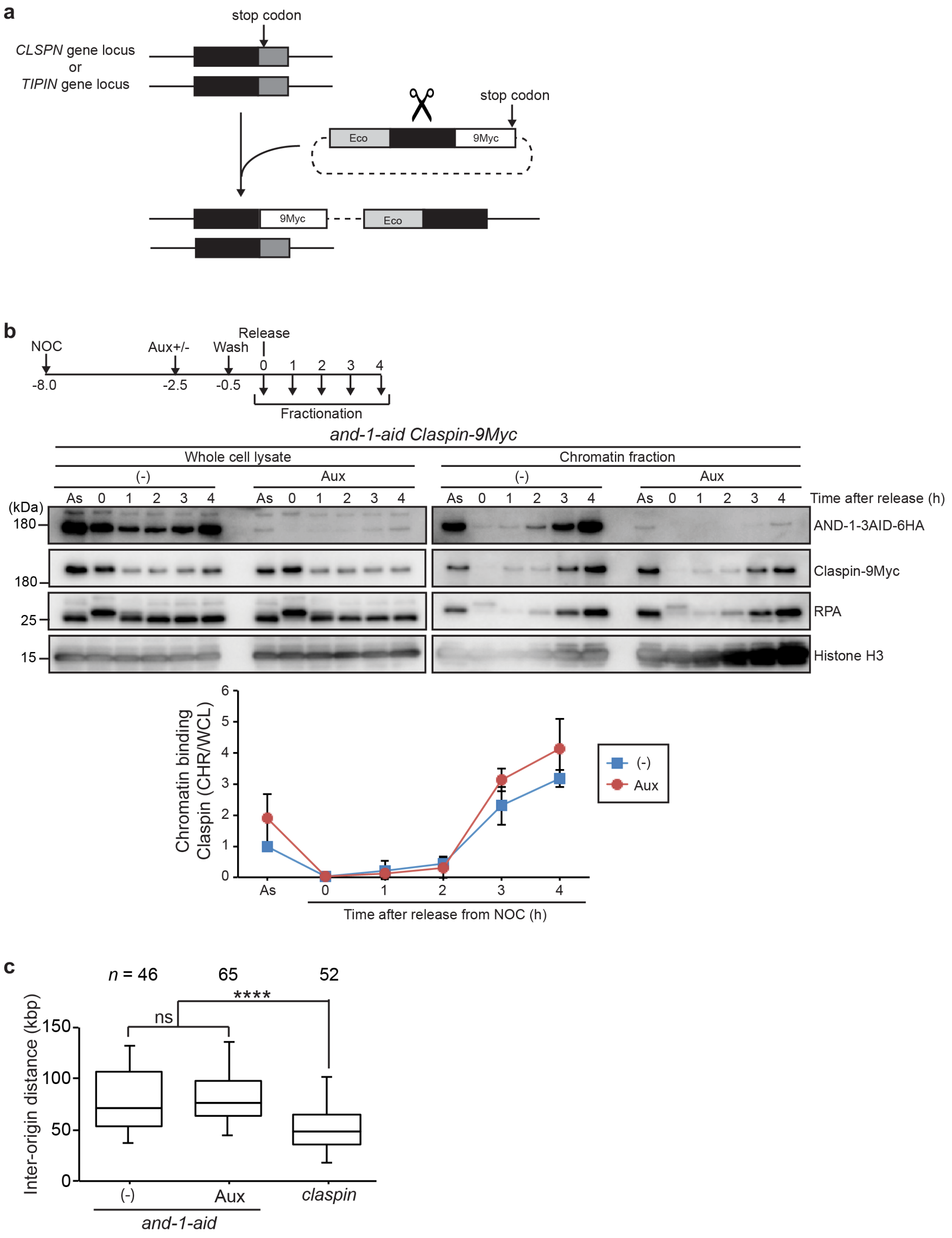

Supplementary Figure 2. Chromatin binding of Claspin in AND-1 depleted cells and inter-origin distance in AND-1-depleted and Claspin-inactivated cells.

a Schematic representation of the flip-in constructs for *CLSPN-9Myc* and *TIPIN-9Myc*. The *CLSPN* gene encodes Claspin. Black boxes indicate exons containing stop codons of each genes, and "Eco" indicates drug resistance gene markers. **b** Upper panel: Presentation of experimental set-up and immunoblot of total lysate and chromatin fraction from *and-1-aid Claspin-9Myc* at indicated time points. Blotting was performed to address AND-1, Claspin, RPA, Histone H3. Bottom panel: Quantification of chromatin bound Claspin in control and AND-1 depleted cells. Ratio of chromatin bound/versus total Claspin-9Myc. Measured chromatin bound Claspin-9Myc amount normalized to the total Claspin-9Myc in the cell lysate of two independent experiments were averaged and plotted. Error bar represents SDM obtained by two independent experiments. **c** Inter-origin distance measured by molecular combing in control, AND-1 depleted and conditional *claspin* mutants. The number n of molecules analyzed from one experiment is included in the graph. Middle line = median; box = 25th and 75th percentiles; bars = 5th and 95th percentiles. *P* values were calculated by Student's t-test. **** indicates a P value ≤0.0001.

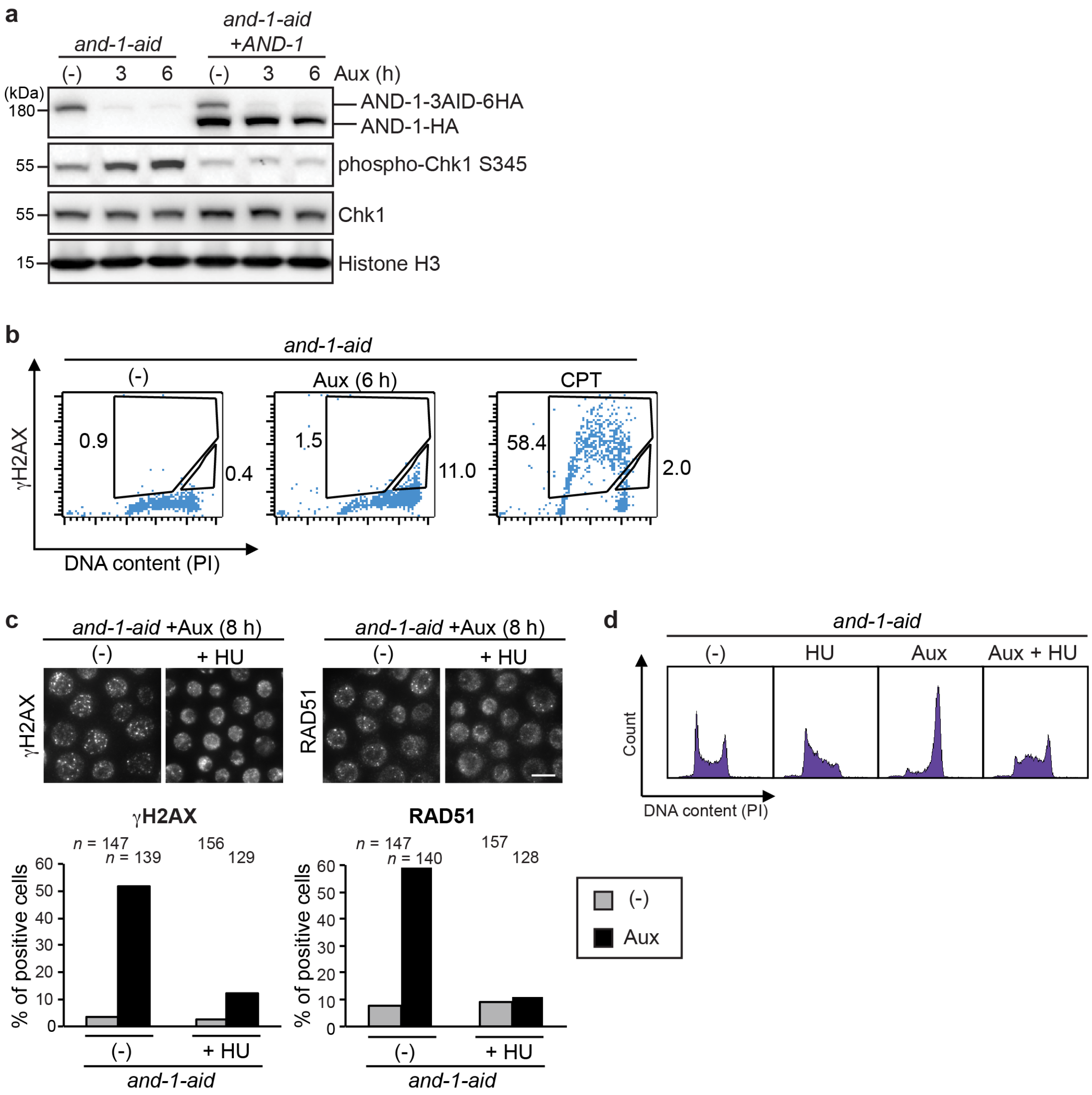

Supplementary Figure 3. DSBs accumulate in AND-1 depleted cells after completion of bulk replication.

a Total cell lysates were prepared from cells with indicated genotype at the indicated time points and analyzed by Western blotting. **b** *and-1-aid* cells were cultured with Auxin or 10 µM CPT for 5 min, and incubated with Propidium Iodide (PI). Cells with DSBs were detected by immunostaining with anti-γH2AX antibody. **c** *and-1-aid* cells were cultured with Auxin for 8 h in the absence or presence of 100 µM HU.

γH2AX- and RAD51-foci were visualized by immunostaining with anti-γH2AX and anti-RAD51 antibodies. Scale bars represents 10 μm. n represents the numbers of cells analyzed in the indicated conditions. **d** Cell cycle distribution of *and-1-aid* cells after treatment with or without Auxin and HU for 8 h.

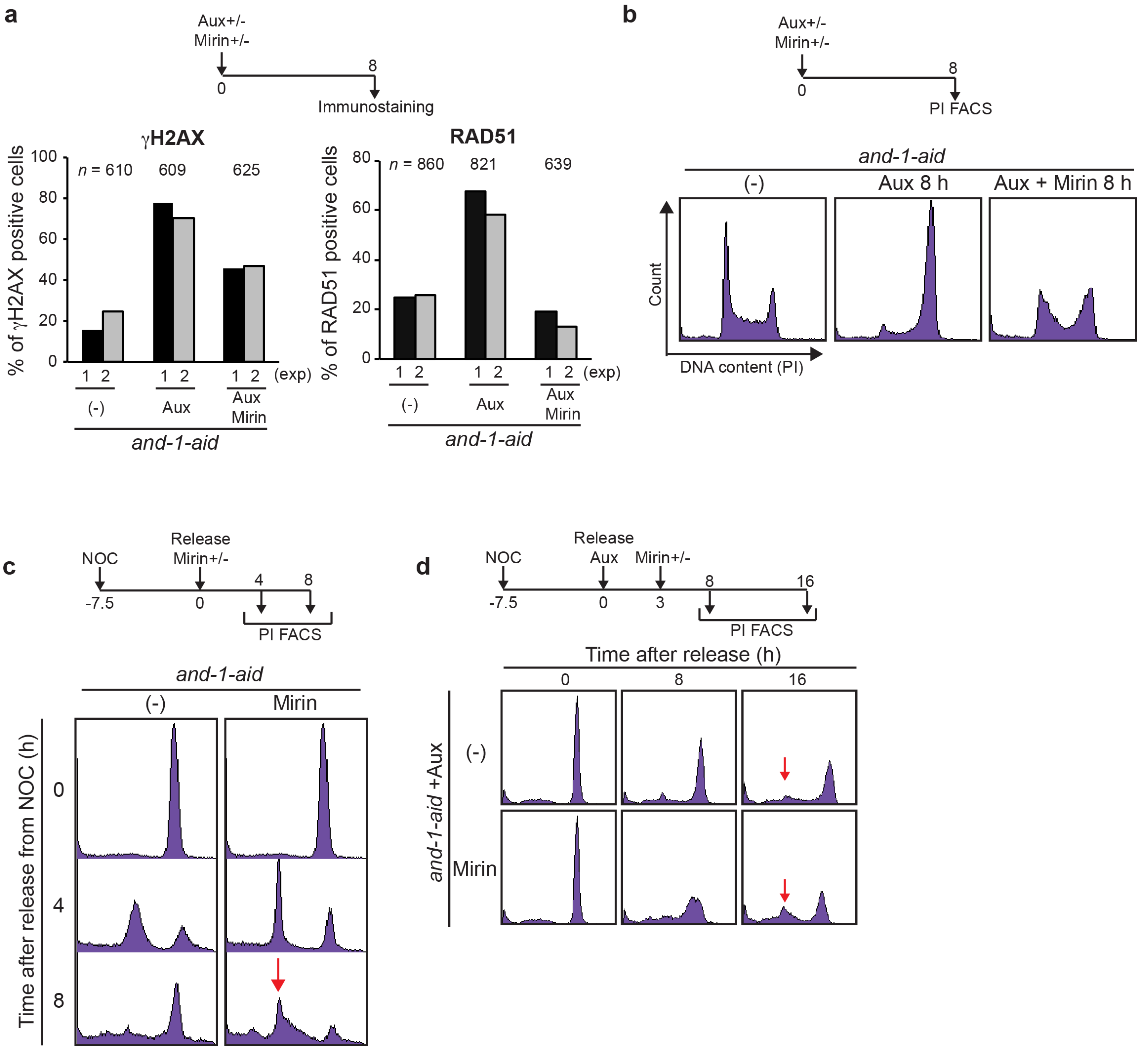

Supplementary Figure 4. Mirin effects on RAD51, γH2AX foci and cell cycle distribution.

a *and-1-aid* cells were incubated with Auxin with or without Mirin for 8 h. γH2AX foci and RAD51 foci were visualized by immunostaining with specific antibodies. Results of two experiments are shown. n represents the number of cells analyzed in the two experiments. **b** Cell cycle distribution of *and-1-aid* cells treated with Auxin with or without Mirin for 8 h. Cells were stained with propidium iodide (PI), and DNA content was analyzed by flow cytometry. **c** Cell cycle distribution of *and-1-aid* cells treated with

or without Mirin after nocodazole (NOC) arrest and release. Cells were harvested at indicated time points and stained with propidium iodide (PI). DNA content was analyzed by flow cytometry. **d** Cell cycle distribution by PI FACS of *and-1-aid* cells treated with or without Mirin after nocodazole (NOC) arrest and release. Mirin was added 3 hours after addition of Auxin. Auxin was added immediately after the release from Nocodazole arrest.

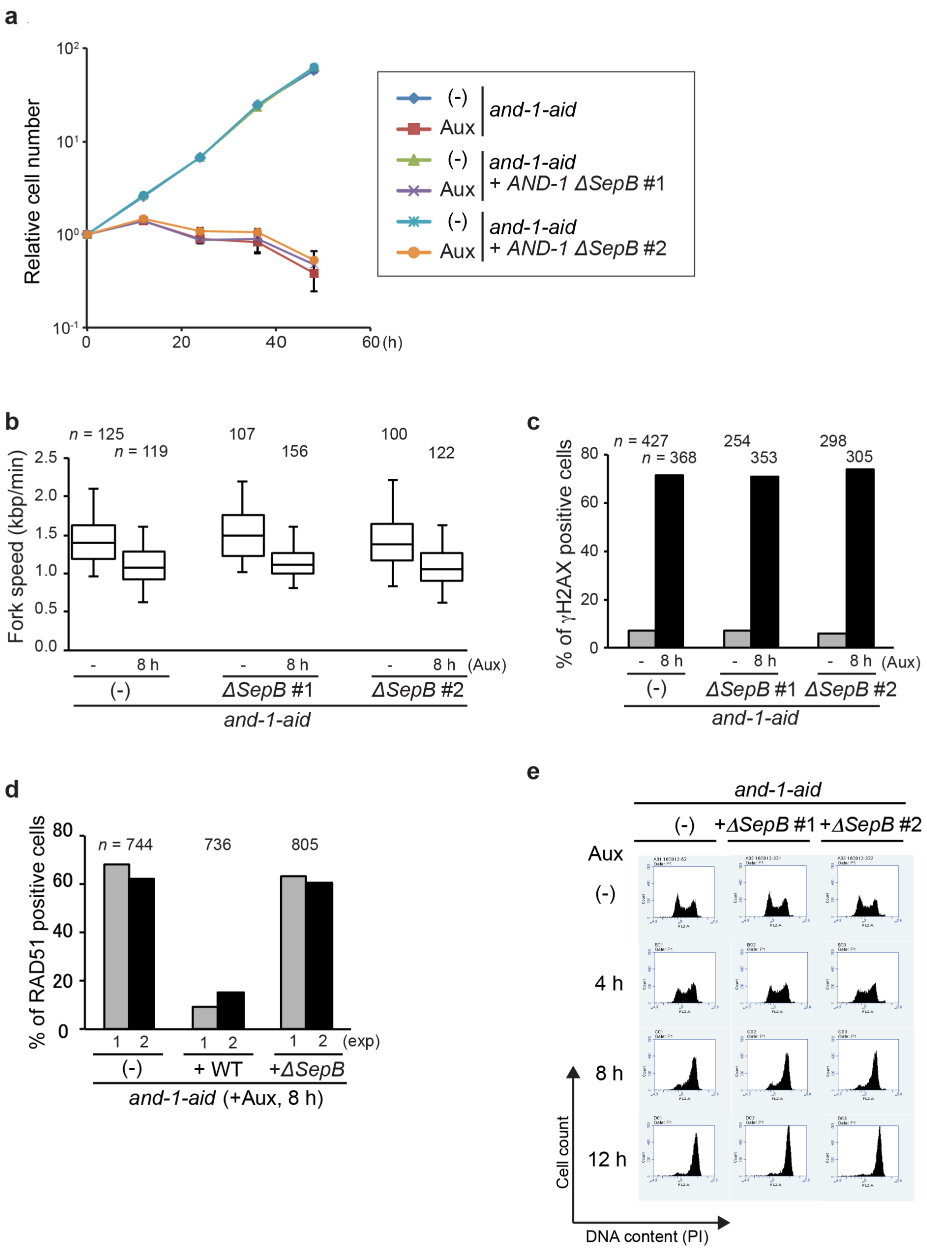

Supplementary Figure 5. The SepB domain of AND-1 is required for proliferation, normal replication fork speed and genome integrity. **a** Growth curves of *and-1-aid* cells expressing AND-1 variant with deletion of the SepB domain in the presence or absence of Auxin. 10^5 cells were inoculated in 1 mL of medium and passaged every 12 h. Error bars represent SDM obtained from three independent experiments. **b** *and-1-aid* cells

expressing or not AND-1 variants truncated for the SepB domain were cultured with or without Auxin for indicated times, and DNA replication elongation rates were calculated as CldU fiber length divided by pulse-labeling time. n indicates the number of fibers analyzed for the indicated condition and genotype. Middle line = median; box = 25th and 75th percentiles; bars = 5th and 95th percentiles. **c-d** *and-1-aid* cells expressing or not AND-1 variant truncated for the SepB domain were incubated with Auxin for 8 h and γH2AX and RAD51 foci were visualized by immunostaining with specific antibodies. n represents the numbers of cells analyzed. **e** *and-1-aid* cells *and-1-aid* cells expressing or not AND-1 variant truncated for the SepB domain were incubated with Auxin for indicated times, stained with propidium iodide (PI), and DNA content was analyzed by flow cytometry.

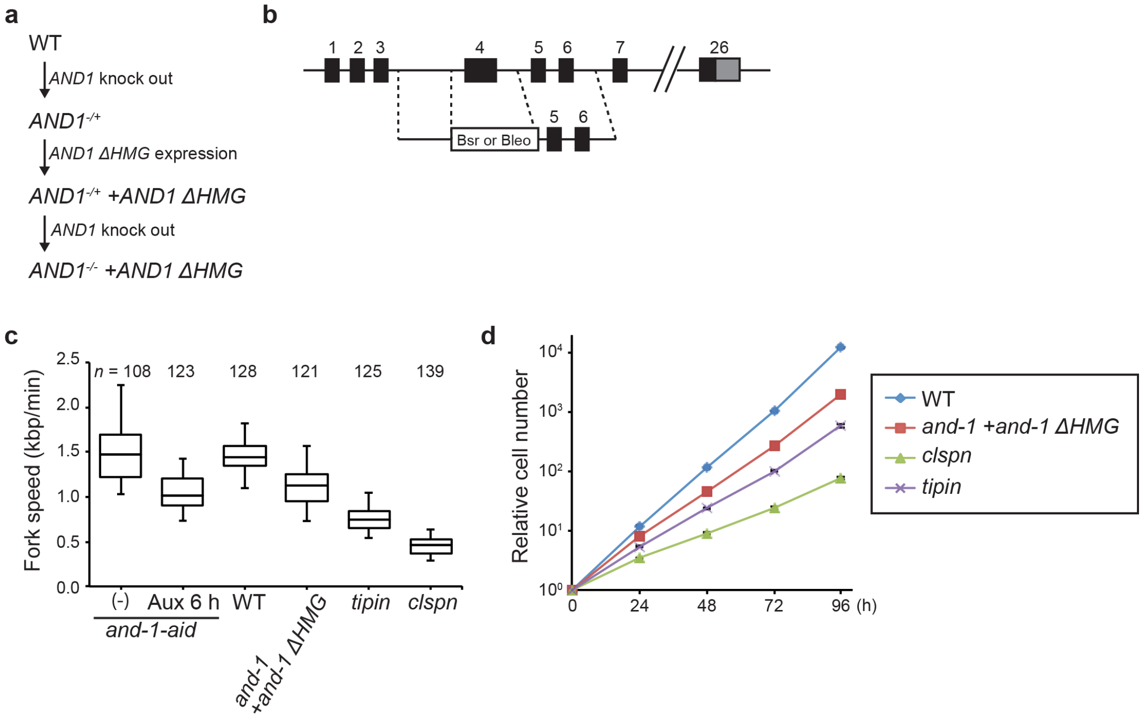

Supplementary Figure 6. Effects of the HMG domain of AND-1 on proliferation and fork speed and comparison with *tipin* and *claspin* mutants.

a Flow chart of *and-1-aid +and-1 ΔHMG* cell's establishment. **b** Schematic representation of *AND-1* gene locus and knock out construct. Black boxes indicate exons, and "Bleo" and "Bsr" indicate drug resistance markers. **c** DNA replication elongation rates of cells with indicated genotypes; constitutive *tipin* and *claspin* knockout cells were used. DNA replication elongation rates, derived from the lengths of CldU tracks (only the ones clearly connected with IdU tracks were considered), are shown. Middle line = median; box = 25th and 75th percentiles; bars = 5th and 95th percentiles. M indicates median values, n, the number of fibers analyzed in each condition. Similar trend was observed in independent experiment. **d** Growth curves of cells with indicated genotypes; here constitutive *tipin* and *claspin* knockout cells were used as controls. 10^5 cells were inoculated in 1 mL of medium and passaged every 24 h. Error bars present SDM obtained from three independent experiments.

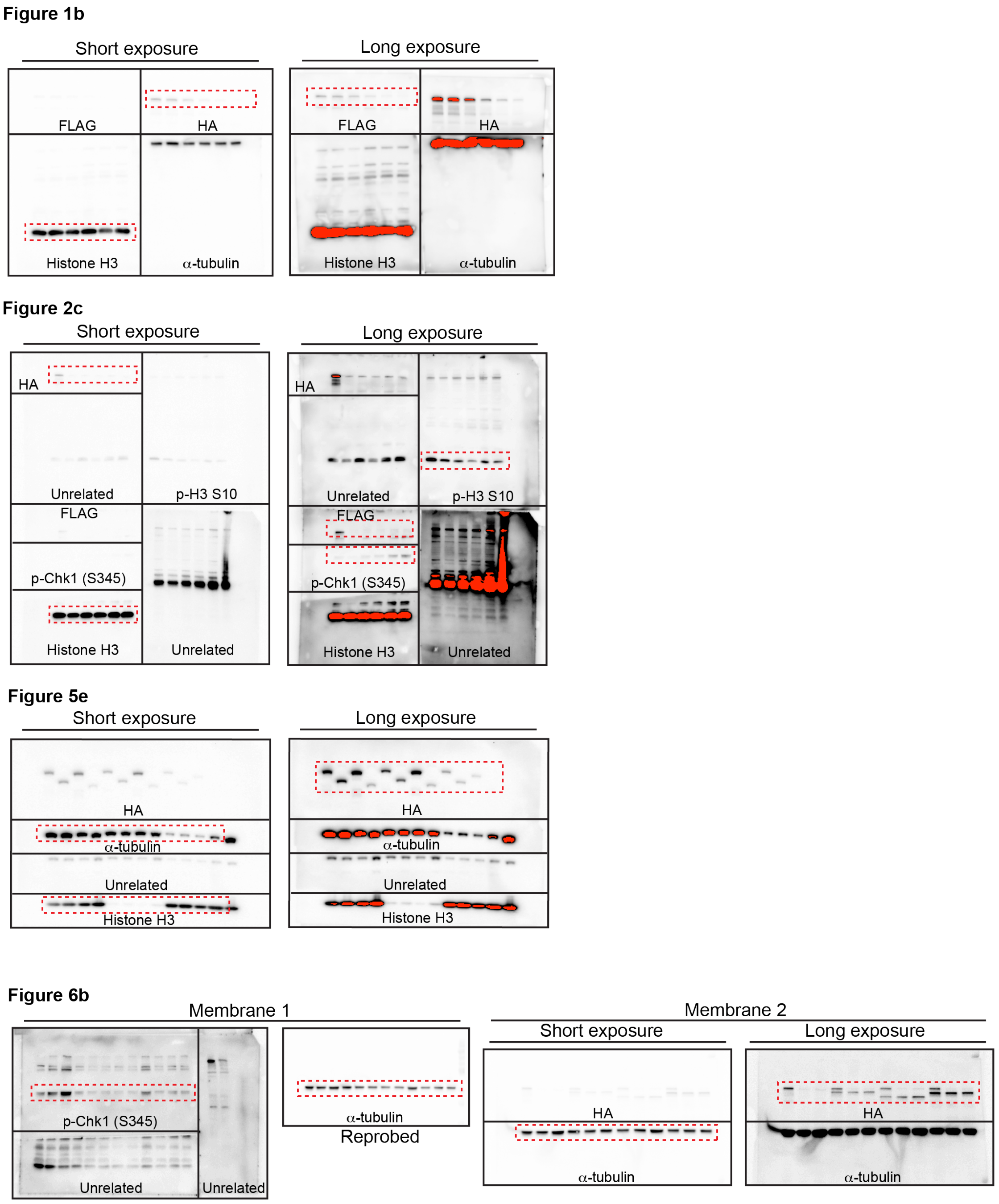

Supplementary Figure 7. Uncropped Western blots corresponding to different panels. Boxes with dashed lines in red color indicate the areas cropped and used in main figures.
